# Efficacy of functional microarray of microneedles combined with topical tranexamic acid for melasma: A randomized, self-controlled, split-face study

**DOI:** 10.1097/MD.0000000000006897

**Published:** 2017-05-12

**Authors:** Yang Xu, Renyan Ma, Juliandri Juliandri, Xiaoyan Wang, Bai Xu, Daguang Wang, Yan Lu, Bingrong Zhou, Dan Luo

**Affiliations:** a Department of Dermatology, The First Affiliated Hospital of Nanjing Medical University, Nanjing; b Suzhou NanoMED skincare Inc, Suzhou, China.

**Keywords:** melasma, microneedle, tranexamic acid

## Abstract

To evaluate the efficacy of a functional microarray of microneedles (MNs) plus topical tranexamic acid (TA) for melasma in middle-aged women in China.

Thirty female subjects with melasma were enrolled in this study. The left or right side of the face was chosen randomly to be pretreated with a functional microarray of MNs, followed by topical 0.5% TA solution once per week for 12 weeks. The other half-face was the control, treated with a sham device plus topical 0.5% TA solution. At baseline and at weeks 4, 8, and 12 of treatment, clinical (photographic) evaluations and parameters determined by Visia were recorded. At baseline and week 12, patient satisfaction scores and the biophysical parameters measured by Mexameter were also recorded. Side effects were evaluated at baseline and at the end of the 12 weeks.

In total, 28 women (93.3%) completed the study. The brown spots’ scores measured by Visia were significantly lower on the combined therapy side than on the control side at 12 weeks after starting treatment; there was no significant difference between sides at 4 or 8 weeks. After 12 weeks, melanin index (MI) decreased significantly in both 2 groups, and the MI was significantly less on the combined side at week 12. Transepidermal water loss, roughness, skin hydration, skin elasticity, and erythema index showed no significant differences between 2 sides at baseline, 4, 8, and 12 weeks after treatment. Physicians’ evaluations of photographs showed better results at week 12 with combined therapy: >25% improvement was observed in the MNs plus TA side in 25 patients, and in the TA side in only 10 patients. Subjective satisfaction scores on both sides increased significantly. The participants were more satisfied with the results of the combined therapy side than the control side. No obvious adverse reactions were observed throughout the study.

Combined therapy with a functional microarray of MNs and topical TA solution is a promising treatment for melasma.

## 1 Introduction

Melasma is an acquired disorder of melanogenesis appearing as localized and chronic hypermelanosis of the face. Its prevalence varies from 5.9% to 35% in different regions of the world.^[1]^ Melasma has a significant impact on appearance, causing psychosocial and emotional distress, and reducing the quality of life of affected patients. Because the pathogenesis of melasma is incompletely understood, the effects of topical drugs and lasers are often less than satisfactory.^[2]^ A number of lasers and light sources have been evaluated for the treatment of melasma, but the risk of postinflammatory pigmentation (PIH) and the possibility of hypopigmentation have limited their potential clinical usage.

Tranexamic acid (TA), a plasmin inhibitor, is reported to improve melasma when administrated orally^[3,4]^ or injected locally.^[5]^ Long-term oral administration of TA may be associated with gastrointestinal adverse effects and an increased risk of thrombosis.^[6]^ The effectiveness of topical TA is debated and doubted, as the water-soluble nature of TA limits its transepidermal absorption. A better and less invasive method is required to enhance absorption of topical TA and replace the need for repeated painful intradermal injections.

Microneedles (MNs) can provide a minimally invasive way for transdermal delivery.^[7,8]^ They have been demonstrated to enhance transdermal delivery of dyclonine,^[9]^ insulin,^[10]^ and other cosmetic products.^[11]^ A functional microarray of MNs is a new type of MNs, which consists of 36 MNs (250 μm in height) over an area of 5 × 5 mm.^[9]^ The MN depth is similar to that of the epidermis, so the microarray does not penetrate the dermis and is therefore painless. The diameter of the tip of each needle is only 80 nm, which takes this skin penetration technology 1 step closer to an almost noninvasive level. In this study, we evaluated the effectiveness and possible side effects of topical TA for melasma with or without pretreatment using a functional microarray of MNs.

## 2 Methods

### 2.1 Participants

**Figure 1:**
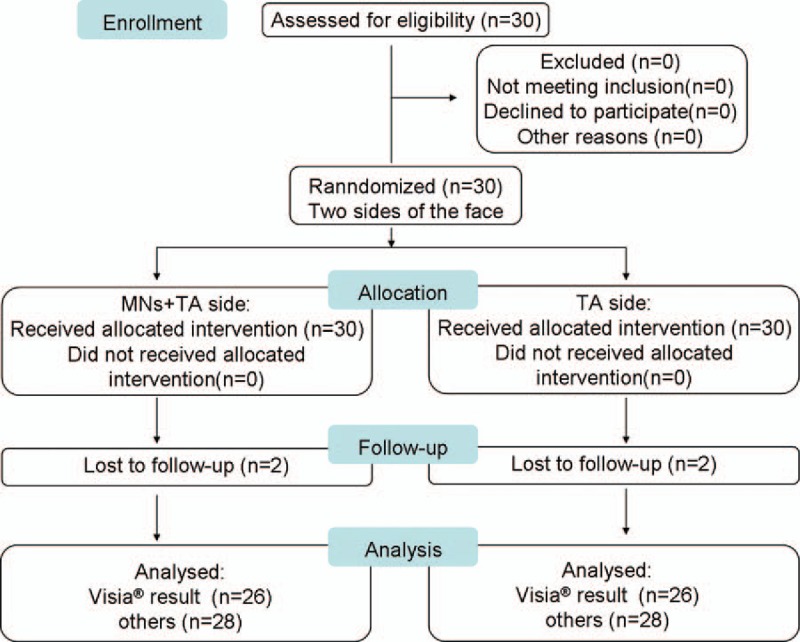
Participant flow diagram; 30 patients met eligibility criteria of a diagnosis of melasma.

Figure 1 shows the participant flow diagram. A total of 30 females with melasma (age range, 20–50 years) with Fitzpatrick skin type III to IV were enrolled from the Dermatology Department of the First Affiliated Hospital of Nanjing Medical University in Nanjing, China, from January 2015 to May 2015. The study protocol was approved by the Institutional Research Committee of the First Affiliated Hospital of Nanjing Medical University. All patients provided written informed consent.

The key inclusion criterion was a clinical diagnosis of melasma by 2 independent senior physicians. Subjects were not included if they had heart, liver, or kidney disease; diabetes; mental illness; a hypercoagulative disorder; or dermatitis on the face. Women who were pregnant, taking oral contraceptives or TA, or treated for melasma within 4 weeks prior to the study were also excluded.

The sample size was estimated. With an estimated efficacy rate in the combined treatment group of 60%, an alpha of 0.05, and an expected error value of 0.05, about 30 samples should be included into each group.

### 2.2 Treatment protocol

A functional microarray of MNs (Nanomed Device Inc, Suzhou, China) described previously^[9]^ was used to facilitate the transdermal absorption of topical TA. The microarray is connected to a pen-like vibrator, which causes the microarray to penetrate the skin with a frequency of 3000 times per minute. The left or right side of the face was chosen randomly as the treated side and the other side as the control. The sides were pretreated with the microarray of MNs or a sham device, respectively. First, 0.5% TA injection solution (Jiening, Changchun Tiancheng Pharmacy Co., Ltd., Changchun, China) was applied all over the face to keep the skin wet. The pen-like vibrator was then moved smoothly and slowly on the surface of the skin, allowing the MN or sham device to stay at the same location for approximately 3 s. During the whole process, 0.5% TA solution was repeatedly applied to keep the skin surface wet until the entire treatment was finished. After completing the treatment, both sides of the face were covered with gauze soaked with the 0.5% TA solution for another 10 min. This treatment was repeated once per week for a total of 12 weeks. All patients were advised to avoid excessive sun exposure and avoid scrubbing the face while cleaning the face.

### 2.3 Outcome measures

#### 2.3.1 Biophysical evaluations

At baseline and at 4, 8, and 12 weeks after beginning treatment, photographs of both sides of the face were taken by Visia (Canfield Scientific, Inc, New York, NY), and scores of brown spots on each side of the face were recorded. In vivo erythema, melanin, transepidermal water loss (TEWL), elasticity, skin surface roughness, and hydration were measured with the corresponding probes of the MPA9 (Courage & Khazaka Electronic GmbH, Cologne, Germany). All measurements were taken after subjects had undergone an acclimatization period of at least 20 min in an air-conditioned room under standardized conditions (22–25°C, 50% humidity). Each measurement was obtained 3 times, and the mean values for each half of the face were compared.

#### 2.3.2 Blinded clinical assessment

Two blinded physician observers evaluated the clinical improvement (compared with baseline) by photographs taken of both sides of the face at weeks 4, 8, and 12 after beginning treatment. The improvement score was determined using a well-established grading scale, in which 0 = <25% (minimal) improvement, 1 = 26% to 50% (fair) improvement, 2 = 51% to 75% (good) improvement, 3 = 76% to 90% (excellent) improvement, or 4 = 91% to 100% improvement (clear skin).

#### 2.3.3 Subjective satisfaction scale

At the end of the 12-week treatment period, the participants were asked to evaluate their satisfaction with the effects on both sides of the face using this scale: 1 = not satisfied, 2 = partially satisfied, 3 = satisfied, or 4 = very satisfied. Adverse events, including erythema, scaling, erosion, itching, and burning, were recorded at each visit.

### 2.4 Statistical analysis

We conducted all statistical analyses using the SPSS software version 18.0 (SPSS Inc, Chicago, IL). Measured data are shown as mean ± standard deviation. Analysis of variance was used to compare brown spot scores at different times in each group. Independent samples *t* tests were used to compare data between the 2 groups at different times. Statistical significance was assumed for a *P* < .05.

## 3 Results

Of the 30 female patients with melasma, the mean age was 38.63 ± 7.11 years. All 30 women had Fitzpatrick skin type III or IV. A total of 28 patients (93.3%) concluded the study; 2 women were withdrawn from the study, as personal reasons prevented them from adhering to the study protocol. Among the 28, 1 did not attend the week 4 follow-up visit, and 1 did not attend the week 8 visit. Thus, Visia measurements and assessments by blinded observers every 4 weeks were evaluated in only 26 patients, whereas MPA9 measurements and patient's satisfaction scores were assessed in 28 patients.

**Figure 2:**
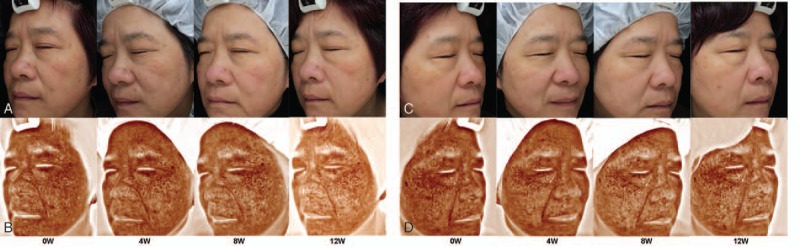
Representative clinical photographs of 2 sides of the face at baseline and at weeks 4, 8, and 12. (A and C) Photographs taken using natural light on the microneedles (MNs) plus tranexamic acid (TA) side and the TA alone side; (B and D) browns spots on the MNs plus TA side and the TA alone side.

As shown in Fig. 1, clinical manifestations of melasma improved at 12 weeks after combined therapy of MNs with TA (Fig. 2A), appearing as lightening and a diminished area of melasma, especially at week 12. By contrast, the pigmentation exhibited no obvious change on the control side (Fig. 2C).

### 3.1 Brown spots

Fewer brown spots were gradually found in the MNs plus TA group (Fig. 2B) compared with the TA alone group (Fig. 2D), which was most obvious at the 12-week assessment. Quantitative analysis confirmed this effect, as determined by the brown spots’ scores measured by Visia. These scores were not significantly different between the 2 groups at the beginning of therapy. A gradual decrease in brown spots’ scores was observed in the MNs plus TA group: 43.33 ± 5.52 before treatment and 36.48 ± 4.84 at week 12 (*P* = .00). Decreased brown spots’ scores were also found in the TA-treated group, but the difference did not achieve statistical significance (41.97 ± 5.83 before treatment and 40.56 ± 5.27 at week 12, *P* = .81). At week 12, the brown spots’ scores were significantly lower in the MNs plus TA group than in the TA alone group (*P* = .00), but no significant differences in scores were observed between groups at week 4 or 8 (Table 1).

**Table 1:**
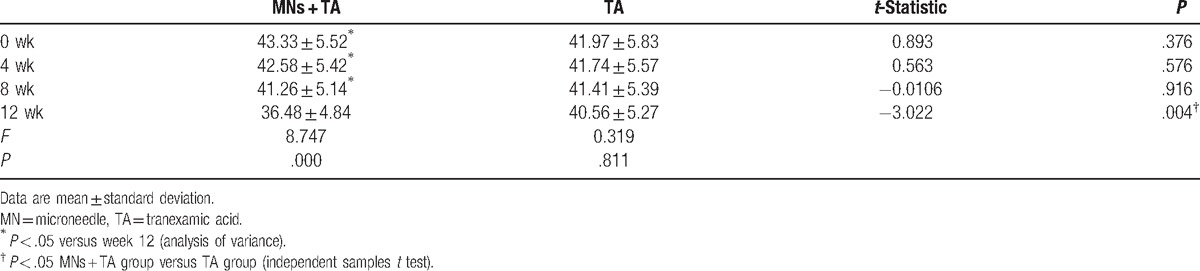
Evaluation of treatment efficacy by Visia measurement.

### 3.2 Melanin index

**Figure 3:**
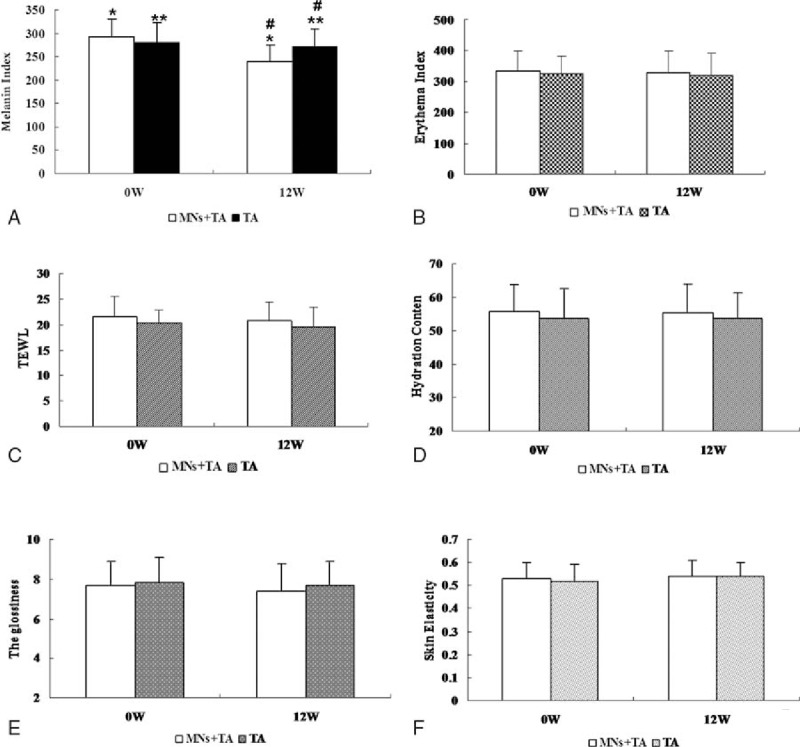
Skin measurements by biophysical evaluation methods obtained at baseline and at 4, 8, and 12 weeks after beginning treatment on both sides, including (A) melanin index, (B) erythema index, (C) transepidermal water loss, (D) hydration, (E) roughness, and (F) overall elasticity (∗*P* = .00, ∗∗*P* = .01, ^#^*P* = .002).

Melanin index (MI) was measured in 28 patients before and after treatment. As shown in Fig. 2A, there was no difference in MI between the MNs plus TA group and the TA alone group at baseline (292.96 ± 38.23 vs. 280.86 ± 41.68, *P* = .26). At week 12, MI decreased significantly in both groups compared with baseline (MNs plus TA: 240.25 ± 36.05, ∗*P* = .00; TA: 272.5 ± 37.54, ∗∗*P* = .01). There was also a significant difference between the 2 therapies at week 12 (^#^*P* = .002, Fig. 3A).

### 3.3 Erythema index

As shown in Fig. 3B, the average erythema index (EI) value exhibited no statistical difference between sides at both the beginning and the end of the entire follow-up period (MNs plus TA: *P* = .05; TA: *P* = .08).

### 3.4 Transepidermal water loss

TEWL was one of the noninvasive indices used to evaluate skin barrier integrity or function. At the beginning of treatment, TEWL was 21.39 ± 4.08 in the MNs plus TA group and 20.25 ± 2.57 in the TA group, whereas at week 12, the TEWL was 20.00 ± 3.53 in the MNs plus TA group and 19.54 ± 3.86 in the TA group. As shown in Fig. 3C, there were no statistically significant differences between groups at any time during therapy, indicating that the functional array of MNs did not produce any changes in the skin's barrier function.

### 3.5 Hydration

Hydration content was also considered as one of the indicators of skin barrier function. As shown in Fig. 3D, hydration was 55.79 ± 8.02 in the MNs plus TA group and 53.64 ± 9.01 in the TA group at the beginning of therapy, whereas at week 12, the hydration content was 55.46 ± 8.72 in the MNs plus TA group and 53.75 ± 7.66 in the TA group. No statistically significant differences were found between the 2 groups at the onset or end of therapy (MNs plus TA: *P* = .31; TA: *P* = .45).

### 3.6 Skin surface roughness

As shown in Fig. 3E, skin surface roughness did not change significantly on either side of the face from before therapy (MNs plus TA: 7.67 ± 1.20; TA: 7.40 ± 1.39) to 12 weeks post-treatment (MNs plus TA: 7.82 ± 1.27, *P* = .12; TA: 7.68 ± 1.23, *P* = .09).

### 3.7 Elasticity

As shown in Fig. 3F, 12 weeks after the treatment, the skin elasticity on both sides of the face (MNs plus TA: 0.54 ± 0.07; TA: 0.54 ± 0.06) was unchanged, compared with the elasticity prior to treatment (MNs plus TA: 0.53 ± 0.07, *P* = .21; TA: 0.52 ± 0.07, *P* = .18), indicating no effect of MNs or TA on skin elasticity at 12 weeks post-treatment.

### 3.8 Subjective assessments

#### 3.8.1 Blinded clinical assessment

**Figure 4:**
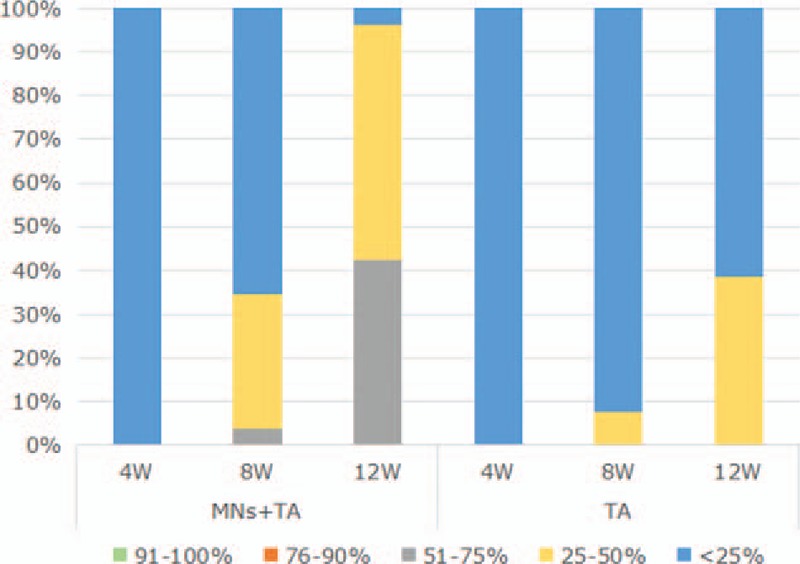
Percentage of subjects with different percentage of improvement from baseline of melasma assessed by blinded physician observers for the microneedles plus tranexamic acid (TA) group and the TA alone group at weeks 4, 8, and 12.

According to the physicians’ evaluations, both sides of the faces in all patients were scored as a 0 at Week 4, which means that the improvement was <25% (Fig. 4). For the MNs plus TA group and TA group, the mean scores at week 8 were 0.38 ± 0.57 and 0.08 ± 0.27, respectively (*P* = .01), whereas at week 12, the mean scores were 1.38 ± 0.57 and 0.38 ± 0.50, respectively (*P* = .00). Significant differences were observed between groups at both weeks 8 and 12.

Improvement was observed from week 8 and gradually increased according to the duration after treatment. More than 25% improvement was observed at week 8 in 9 patients (34.62%) in the MNs plus TA group and only 2 patients (7.69%) in the TA group. Further improvement was found at week 12, with more than 25% improvement noted in 25 patients (96.15%) in the MNs plus TA treatment group and only 10 patients (38.46%) in the TA group (Fig. 4).

#### 3.8.2 Subjective satisfaction scale

During the evaluation period, the subjective satisfaction scores in both groups increased significantly (Table 2). At 12 weeks after treatment, the mean subjective satisfaction score in the MNs plus TA group was 2.75 ± 0.84, which was significantly higher than that of the control TA group (1.61 ± 0.57, *P* = .00).

**Table 2:**
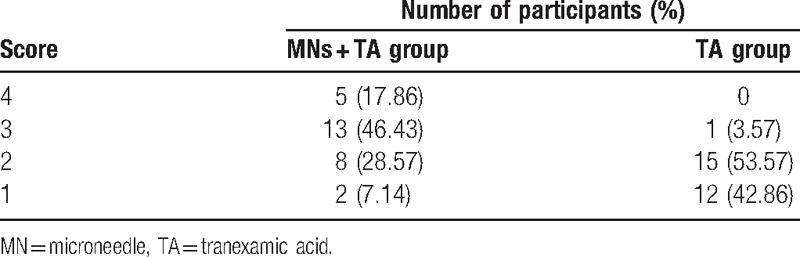
Subjective satisfaction scale scores at week 12.

**Figure 5:**
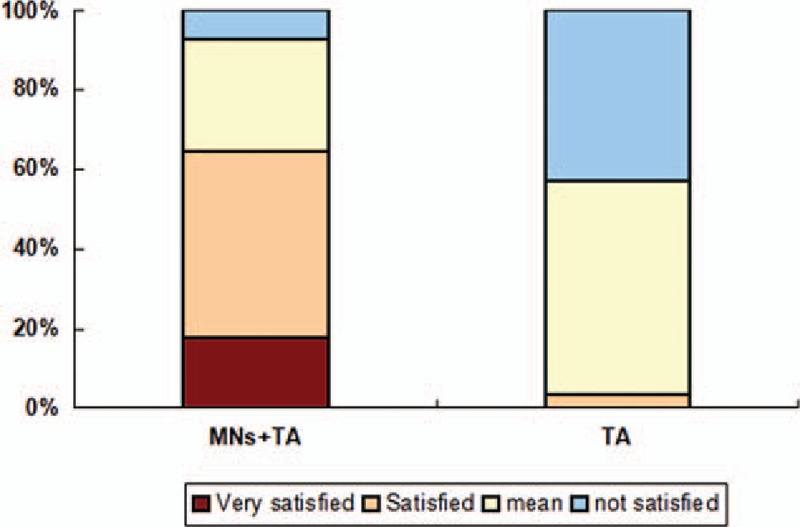
Percentage of patients with different satisfaction score for the microneedles (MNs) plus tranexamic acid (TA) group and the TA alone group at week 12.

At the end of the 12 weeks of treatment, 18 patients (64.29%) in the MNs plus TA group were at least satisfied with the final results (subjective satisfaction scale of 3 or 4). By contrast, only 1 patient (3.57%) was at least satisfied with the final results in the TA group and 53.57% were partially satisfied (score of 2) (Fig. 5).

#### 3.8.3 Side effects

No erosion, ulcer, scaling, itching, or burning was reported at any visit. Just 1 patient was found to have dermatographism after MNs treatment; this disappeared after approximately 20 min, so the treatment was not stopped. Of note, this woman had pressure urticaria for the past 3 years.

## 4 Discussion

Melasma is one of the most common pigment disorders, which typically occurs among women from 30 to 40 years of age with Fitzpatrick skin types III to IV. The cause of melasma remains unknown; however, there are many possible contributing factors, such as genetic predisposition, endocrine dysfunction, use of oral contraceptive pills, pregnancy, exposure to sunlight, improper cosmetics, and excessive scrubbing of the face.^[12]^ The underlying mechanism of melasma is thought to be related to hyperproliferation of melanocytes, increased synthesis of melanin, local inflammation, and impaired skin barrier function.^[12]^ Until now, melasma has lacked specific, effective therapy.

TA is one of the treatment strategies for melasma that has been a focus of attention in recent years. Potential mechanisms for its effectiveness include the following: inhibition of melanocyte proliferation^[13]^; inhibition of melanin synthesis in melanocytes^[14]^; accelerated recovery of impaired skin barrier function^[15]^; reduced number of blood vessels in the dermis^[16]^; and reduced number of mast cells in the dermis.^[16]^

Oral administration of TA was reported in several studies to be effective for melasma. After oral TA for 4 months, 89.7% of Korean patients had documented improvement,^[17]^ and at a dosage of 250 mg twice daily for a therapeutic period of 6 months, TA was effective in 64.8% of Chinese patients.^[3]^ Furthermore, a preliminary study of 2 tablets of compounded TA administered 3 times per day reported that improvement occurred in 85% of patients in 4 weeks, 97% in 8 and 12 weeks, and 100% in 16 weeks.^[4]^ TA is a synthetic lysine analog that inhibits plasmin activity, which has been used as an antifibrinolytic agent for over 30 years. The side effects of oral TA remain a concern. In addition to the possibility of gastrointestinal discomfort, headache, and hypomenorrhea, the most important potential risk is systemic thrombosis formation.^[6]^

To avoid systemic administration of TA, localized microinjection of TA has been used for melasma. It was reported to be effective,^[5]^ with statistically significant reduction in the Melasma Area and Severity Score (MASI) at 8 and 12 weeks, but the whole process requires local anesthesia to reduce the pain accompanying the multiple injections.

The results of topical use of TA for excessive pigmentation have been conflicting. In a report by Yuan et al, 5% TA solution was effective in decreasing the MI value in human damaged skin, suggesting that melanism from ultraviolet B irradiation can be rectified by TA.^[15]^ Topical 3% TA solution twice per day for 12 weeks was likewise found to be as effective as a topical solution of 3% hydroquinone plus 0.01% dexamethasone.^[18]^ However, another study showed that lightening of pigmentation by 5% TA gel for 12 weeks was neither superior to nor different from treatment with its vehicle.^[19]^ Debate exists whether topically applied water-soluble TA is transdermally absorbed through the lipid-soluble surface of the skin.

Strategies to enhance transdermal delivery of TA have been developed. Fractional carbon dioxide (CO_2_) laser was reported to increase TA absorption in porcine skin,^[20]^ but when applied to humans, the risk of PIH caused by the fractional CO_2_ laser itself should be considered. MNs can provide a minimally invasive means of painless delivery of therapeutic molecules through the skin barrier with precision and convenience. Physically opening microtunnels for drug delivery with MNs involves a different mechanism of action than lasers and is not accompanied by the risk of PIH. Increased MN-assisted transdermal delivery has been demonstrated for a variety of compounds, including calcein,^[8]^ methyl nicotinate,^[21]^ fluorescein isothiocyanate-labeled Dextran,^[7]^ bovine serum albumin,^[22]^ insulin,^[10]^ plasmid DNA,^[23]^ and nanospheres.^[24]^

In our study, we found that topical TA plus pretreatment with microarrays of MNs produced significantly better effects on melasma than topical TA alone after 12 weeks of therapy, with lower brown spots scores, lower MI values, greater improvement according to blinded physician assessments, and superior patient satisfaction. Pretreatment with MNs enhanced the effects of topical TA, and the whole process was painless, being much more acceptable than local injections.

After 6 to 8 weeks of combined therapy, pigmentation within patches of melasma was diminished in a punctate pattern and the edge of the patches began to appear vague. We theorize that this phenomenon may be related to inhibition of melanocyte proliferation and melanin production. Reduced transportation of melanin from the dendritic protrusions of melanocytes to keratinocytes led to the spotty whitening effects within the pigmented patch. Further experiments are required to establish the validity of this theory.

Skin barrier function is decreased in areas of melasma. In the present study, we noted no significant changes in TEWL between before and after therapy on either side of the face. The application of MNs once per week did not cause long-term impairment of barrier function. Although it has previously been reported that TA could be helpful for the recovery of barrier function, no such effect was found in our study. This may be related to the less direct absorption of TA solution and the use of MNs only once per week.

**Figure 6:**
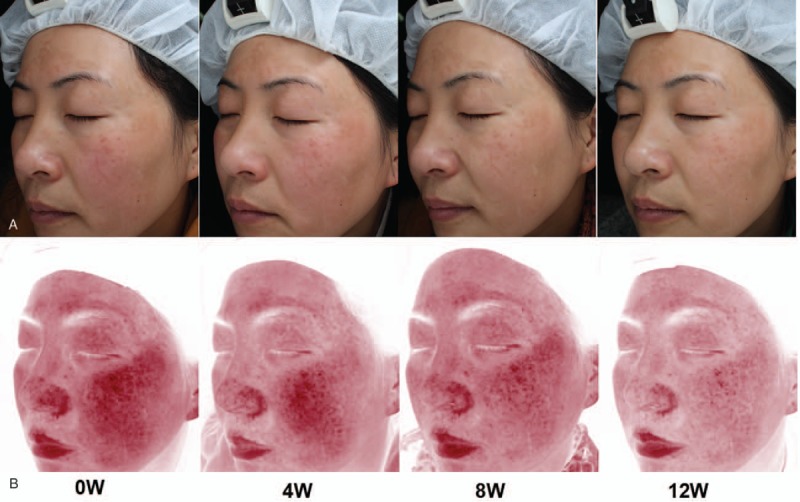
Clinical observations showing improvement of melasma (A) accompanied by diminished red patch (B) in microneedles plus tranexamic acid side at weeks 4, 8, and 12.

No differences in EI were found between the 2 treatments in our study. However, accompanying the improvement of melasma on the combined therapy side (Fig. 6A), we found a decreased area of red patches by Visia in some patients (Fig. 6B), which may be explained by less proliferation of blood vessels due to TA. It is known that plasmin plays an important role in angiogenesis, as it converts extracellular matrix-bound vascular endothelial growth factor into freely diffusible forms, whereas TA suppresses angiogenesis and also inhibits neovascularization induced by basic fibroblast growth factor. Thus, TA could reduce erythema and the number of blood vessels. Additional studies involving the use of more samples, as well as pathological examination, are required to establish the effect of combined MNs plus topical TA on vascularity.

Although topical TA alone generally did not exhibit significant effects in our study, the MI values in both groups decreased significantly after 12 weeks of therapy. This may have been attributed to reduced sun exposure and scrubbing of the face, which we recommended to all patients at the onset of the 12-week treatment period. Thus, skin care was probably also important for melasma therapy. There was a significant difference in the MI between the 2 treatment groups at week 12, indicating that combined therapy was more effective than TA alone.

One limitation of our study was that no TA cream is available in China, so we used a TA injection solution instead. In our study, it was important to keep the skin wet using the TA solution during treatment and covering the area with a gauze soaked with 0.5% TA solution for another 10 min after treatment. In the present study, we did not use the MASI (a common index for the clinical evaluation of melasma) because it is composed of scores of areas and severity of the whole face, so it cannot be used for half-face assessments. Perhaps a more specific scoring system for split-face studies could be developed for future usage. Another limitation of our study was the observation period of only 3 months; long-term follow-up is required to confirm the efficacy results and to evaluate relapse rates. Histological evaluation would be useful in the future to further verify the clinical effects, although it is difficult to expect patients to agree to have a biopsy obtained from their face.

## 5 Conclusions

The above findings suggest that pretreatment with a functional microarray of MNs can significantly increase the effectiveness of topical TA in treating melasma, and the combined therapy is safe and painless, without obvious side effects. Therefore, our study has broad scientific impact by establishing a novel regime for the treatment of melasma.
